# Supplementary material for: Development of a computer-aided design software for the quantitative evaluation of aesthetic damage
Source: PLoS One. 2019 Dec 18;14(12):e0226322. doi: 10.1371/journal.pone.0226322 (PMC6919621; doi:10.1371/journal.pone.0226322)
Supplement: S1 Code site — 1. (ZIP) [file pone.0226322.s001.zip › [EESC_jr][Projeto]Relatorio_07_21/reports/templates_zip/gentelella-master/vendors/bootstrap-daterangepicker/example/amd/index.html]

A date range picker for Bootstrap


# Configuration Builder

parentEl

startDate

endDate

minDate

maxDate

autoApply

singleDatePicker

showDropdowns

showWeekNumbers

showISOWeekNumbers

timePicker

timePicker24Hour

timePickerIncrement (in minutes)

timePickerSeconds

dateLimit (with example date range span)

ranges (with example predefined ranges)

locale (with example settings)

linkedCalendars

autoUpdateInput

alwaysShowCalendars

opens

right
left
center

drops

down
up

buttonClasses

applyClass

cancelClass

#### Your Date Range Picker

#### Configuration
